# The color of environmental noise in river networks

**DOI:** 10.1038/s41467-023-37062-2

**Published:** 2023-03-28

**Authors:** Tongbi Tu, Lise Comte, Albert Ruhi

**Affiliations:** 1 School of Civil Engineering, Sun Yat-sen University, Guangdong, 519082 China; 2 Department of Environmental Science, Policy & Management, University of California, Berkeley, CA 94702 USA; 3 School of Biological Sciences, Illinois State University, Normal, IL 61790 USA

**Keywords:** Hydrology, Freshwater ecology, Ecology

## Abstract

Despite its far-reaching implications for conservation and natural resource management, little is known about the color of environmental noise, or the structure of temporal autocorrelation in random environmental variation, in streams and rivers. Here, we analyze the geography, drivers, and timescale-dependence of noise color in streamflow across the U.S. hydrography, using streamflow time series from 7504 gages. We find that daily and annual flows are dominated by red and white spectra respectively, and spatial variation in noise color is explained by a combination of geographic, hydroclimatic, and anthropogenic variables. Noise color at the daily scale is influenced by stream network position, and land use and water management explain around one third of the spatial variation in noise color irrespective of the timescale considered. Our results highlight the peculiarities of environmental variation regimes in riverine systems, and reveal a strong human fingerprint on the stochastic patterns of streamflow variation in river networks.

## Abstract (web summary)

The color of environmental noise, or degree of predictability in environmental variation, has important implications for ecosystem conservation and management. This study investigates the patterns and drivers of noise color across the US rivers.

## Introduction

Environmental variation is inherent to natural ecosystems, and consists of deterministic (“signal”) and stochastic (“noise”) components^1^. The direction and degree of temporal autocorrelation in environmental noise, or “noise color”, measures the persistence of stochastic variation in the environment, and has received relatively little attention despite presenting key implications for ecosystems^2,3^. Environmental fluctuations can be purely random (white noise), or autocorrelated (e.g., reddened spectra, reflecting positive autocorrelation where conditions can remain stable for a certain time; or blue spectra, reflecting negative autocorrelation where conditions vary faster than white noise)^4^. The characteristics of environmental fluctuations (including noise color) have important implications for population dynamics, species persistence, and community stability^5–7^. Noise color can also affect the management of natural resources such as water, since autocorrelation structures influence intervals between extreme events (e.g., droughts and floods)^1,8^. Understanding the spatio-temporal patterns in environmental noise color in ecosystems, and how they are controlled by natural and anthropogenic drivers, is thus consequential for natural resource management.

Previous research has shown that marine environments are more reddened than terrestrial ecosystems, likely because large bodies of water buffer variation at short frequencies (e.g., diel swings in temperature)^4^. Freshwater ecosystems such as lakes and ponds are smaller^9^; and are thus likely to be less buffered against short-term variation than oceans. The dendritic spatial structure of rivers and directional flow controlling the movement of energy, matter, and organisms from headwaters to the estuary could also represent a key difference relative to oceans^10–12^. Whereas streams and rivers may present reddened daily flows (from slightly pink to black noise color^1^), flow noise color is expected to change within and across river networks, as drainage area increases and rivers collect water from a larger diversity of landscapes and climates^13^.

Beyond natural biophysical processes, human activities may modify environmental variation in natural environments, potentially “coloring” noise^14^. For example, flow regulation can dampen or exacerbate flow variability driven by hydroclimate^15–19^, and the artificial movement of water from wet to dry seasons or from water-abundant to water-scarce watersheds can cause widespread flow regime stabilization^20,21^. Similarly, urbanization often leads to increased runoff and decreased infiltration, thereby increasing flashiness and changing temporal flow variability^22^. Anthropogenic climate change could also affect the color of environmental noise—both directly (e.g., via increased magnitude and frequency of intermittency and flash floods), or by interacting with anthropogenic processes such as increased water storage to satisfy human needs in the face of water scarcity^15,23,24^.

Finally, another important, yet often overlooked consideration is that environmental noise color may be time-scale sensitive^1,25^. Comparisons of flow noise color at multiple scales (e.g., daily and annual) have been rare in the literature^1^. Given that the dynamics of hydroclimate and human activities can vary at a range of temporal scales^26^ and that short- and long-lived organisms may experience environmental variation in different ways (e.g., as stress or as cues that entrain life histories)^4,26,27^, the structure and drivers of environmental noise color should be examined across a range of ecologically-relevant temporal scales.

In this work, we explore the geography and drivers of flow noise color in river networks. We first examine the spatial patterns of flow noise color at different temporal scales (daily and annual) across streams and rivers in the conterminous United States by spectral methods on 7504 gages with long-term high resolution discharge data. We then quantify the associations and relative importance of a suite of natural and anthropogenic variables (geography, hydroclimate, land-use, regulation by dams). Our analysis demonstrates that the characteristics of environmental fluctuations in riverine networks differ from those previously reported from terrestrial and marine ecosystems, and bear the signature of human activities. These results advance current understanding of stochastic variation in the environment—potentially assisting in the identification, management, and conservation of river ecosystems degraded by hydrologic alteration.

## Results

### Spatial variability in flow noise color

**Fig. 1:**
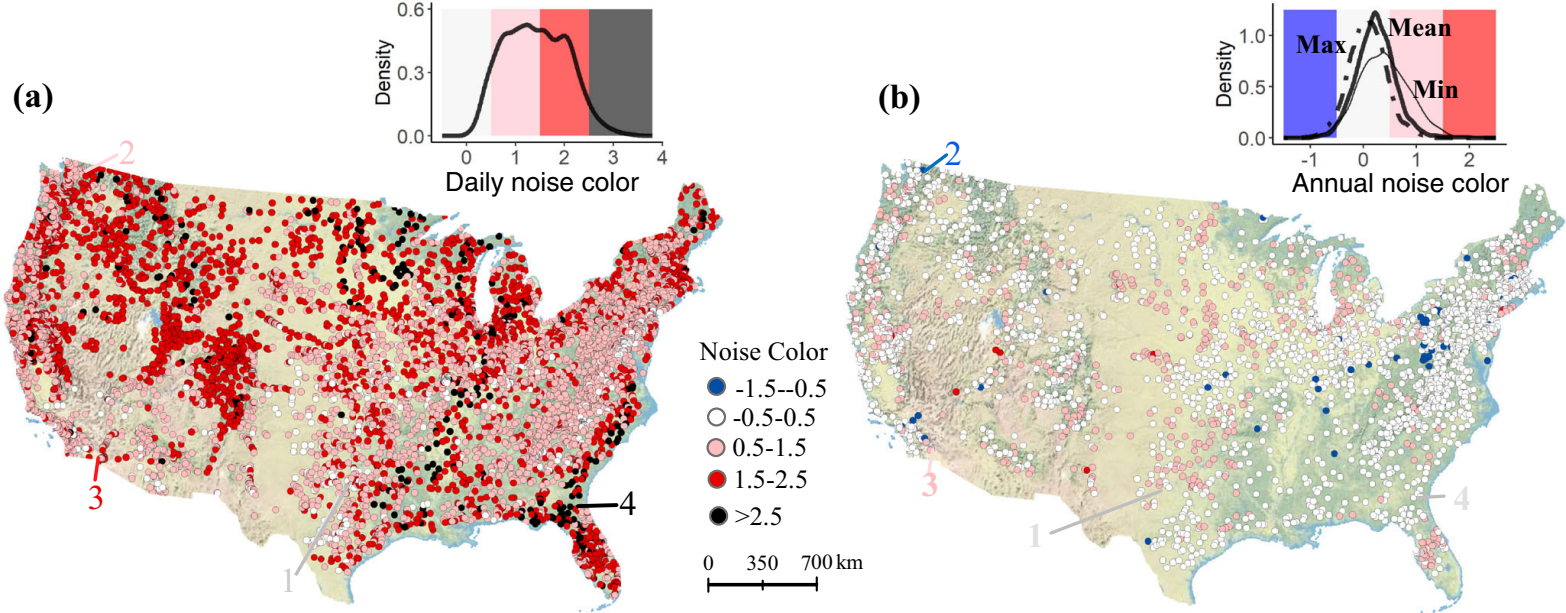
Observed patterns of flow noise color across 7504 (for daily data) and 2594 stream gages (for annual data) across the conterminous United States. In **a** and **b** we show the spatial distribution of daily and annual flow noise color respectively, together with their density distributions (for annual flow noise color, we show the mean, minimum, and maximum annual flow; obtained from the average, minimum, and maximum daily flows in a year). Four different sites are shown, selected to illustrate a range of flow regimes: site 1, USGS gage #08086212, Hubbard Creek in Albany, Texas (white daily and annual noise color); site 2, USGS gage #12178100, Newhalem Creek in Washington (pink daily and blue annual noise color); site 3, USGS gage #10255550, New River in Westmorland, California (red daily and pink annual noise color); and site 4, USGS gage #02228000, Satilla River in Atkinson, Georgia (black daily and white annual noise color). The background of the CONUS credits to Copyright:© 2013 National Geographic Society, i-cubed in ArcGIS® software by Esri.

**Fig. 2:**
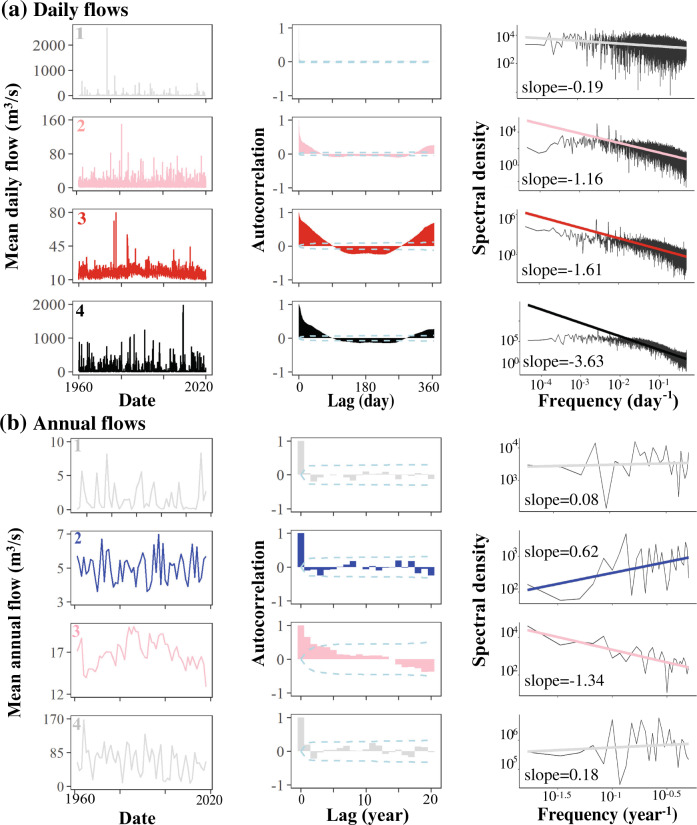
Observed flow regimes and noise color at daily and annual scales. In **a** and **b** we show hydrographs, Autocorrelation Functions (ACF), and spectral density plots for four different sites (shown in Fig. 1), calculated based on daily flow values (**a**), and mean annual flow values (**b**). The spectral density plots show how noise color is calculated from the linear regression of spectral density against frequency (noise color is the slope in opposite sign; see Eq. 3 in Methods). Warmer colors indicate higher ‘memory’–as indicated by significant correlation at longer lags.

We observed a large range of variability in flow noise color across space, and large differences between noise color based on daily versus annual time series. We quantified flow noise color by analyzing the spectrum of frequencies, using long-term records (15–50 years) of mean daily discharge from 7504 gages for daily analyses and 2594 gages for annual analyses. For daily flows, reddened spectra (i.e., positive values) dominated (Fig. 1a), with 48.5% of the gages displaying pink noise colors (i.e., values ranging from 0.5 to 1.5), and 41.0% of the gages displaying red noise colors (i.e., from 1.5 to 2.5). In contrast, only 6.8% of the daily flow time series were categorized as white (0 to 0.5), and 3.7% as black (>2.5). Annual flows presented much whiter spectra, regardless of the metric being analyzed—mean annual flows, maximum annual flows, or minimum annual flows (Fig. 1b). However, some patterns across metrics emerged. While 85.5% of the gages showed white (random) noise for annual maximum flows (median = 0.08), minimum flows showed relatively more persistent variation (57.3% of “white” gages; median = 0.23), and annual mean flows fell in between the two (77.0%; median = 0.39) (Fig. 1b). This result indicates that while flows have high “memory” at short timescales (i.e., from one day to the next), in most of the country wet or dry years do not predict next-year conditions (i.e., the annual mean flow at a high or low magnitude may not be very likely to occur in consecutive years). The observation that minimum annual flows showed more persistent dynamics than maximum annual flows also suggests that extreme low flows (often associated with droughts) are more likely to extend for long time periods, whereas anomalously wet years in a row are uncommon. Moreover, our results indicated that daily flow noise color is a poor indicator of annual flow noise color (Pearson’s correlation coefficient *r* < 0.1). For instance, persistent flow variation (i.e., pink, red, or black) at the daily scale often turned into random (white) or anti-persistent behavior (blue) when analyzed at the annual scale, as illustrated by site 2 in Fig. 2. An illustration of how the noise color was calculated can be found in Fig. 2.

We also found that noise color patterns were almost identical regardless of the option selected for detrending long-term linear trends and seasonal cycles (see Methods for details). Correlation coefficients for noise color estimates based on data that had been transformed using different methods ranged from 0.999 to 1.0. While differences in flow noise color estimates were observed in some gages, mean differences were overall negligible, averaging −0.006 or −0.012 units relative to the “raw” version, depending on the method (Fig. S1).

In order to test for multiple scaling regimes, we further compared daily noise color estimates obtained across a range of timescales (Figs. S2 and S3). We observed a flattening pattern towards the lower frequencies (i.e., longer periods). Across the whole set of 7504 gages, the average change in noise color estimates when comparing “global” slopes to the sub-monthly “local” slopes (i.e., 7–30 days; Fig. S3a) was on average 0.19, and 77.3% of the gages showed decreased values. When comparing local slopes at the seasonal scale (i.e., 30–180 days) to sub-monthly scales (Fig. S3b) that difference was of −0.41 on average, and 77.2% of the gages showed decreased values. These results are consistent with Vasseur and Yodzis^4^, who reported that 60% of analyzed spectra (on a range of environmental time series) flattened at lower frequencies. However, we note here that the existence of multiple scaling regimes does not undermine the representativity of the global scaling regime, as higher frequencies tend to dominate the regression, and the impact of flattening at low frequencies is often trivial to the regression^4^. Moreover, noise color at the global scale was strongly correlated with that obtained at sub-monthly (7–30 days, *r* = 0.90) and intra-annual (7–365 days, *r* = 0.87) scales, indicating that daily noise color extracted from global scales can be a good indicator of the short-term stochastic variation. At the annual scale, the “local” slope of daily noise color still partially captured variation in noise color estimates (Fig. S3c, d). However, at that scale (and any scale longer than annual), annual-scale analyses are preferable, as they are not influenced by the confounding effects of sub-annual autocorrelation in the data. In this vein, at temporal scales larger than a year, noise color based on annual flows was required, as daily noise color at this scale flattened significantly, and linear relationships between the log-transformed spectra against its frequency did not hold for 91% of the gages. Overall, the two scales of analysis (based on daily and annual data) allowed us to explore a broad range of scales, with daily noise color accurately capturing flow patterns that occur at scales shorter than annual, and annual noise color better capturing interannual patterns.

At the daily scale, white noise was mostly found in rivers across the Southwestern U.S. and the Southern Great Plains, which could be explained by dominant flashy hydrologies (i.e., flow regimes in grassland and prairie streams dominated by Hortonian overland flows, which tend to rise and fall quickly after precipitation events) in these regions. Pink noise was found along the West Coast and Appalachia, and the most “reddened” categories (red and black noise colors) were found in clusters across the Rocky Mountains and the Sierra Nevada ranges, the Great Lakes, the South-East coast, and Florida (see Fig. 1 and USGS Hydrologic Unit Codes in Fig. S4). At the annual scale, white noise dominated nation-wide, but pink color was sometimes observed in California’s Sierra Nevada, the Central Plains, and Florida. In turn, blue noise (associated with “whiplash” patterns of anomalously low versus high-flow years) was scattered, with some clustering across the Midwest and Southwest deserts (Figs. 1 and S5).

### Natural and anthropogenic drivers of flow noise color

**Fig. 3:**
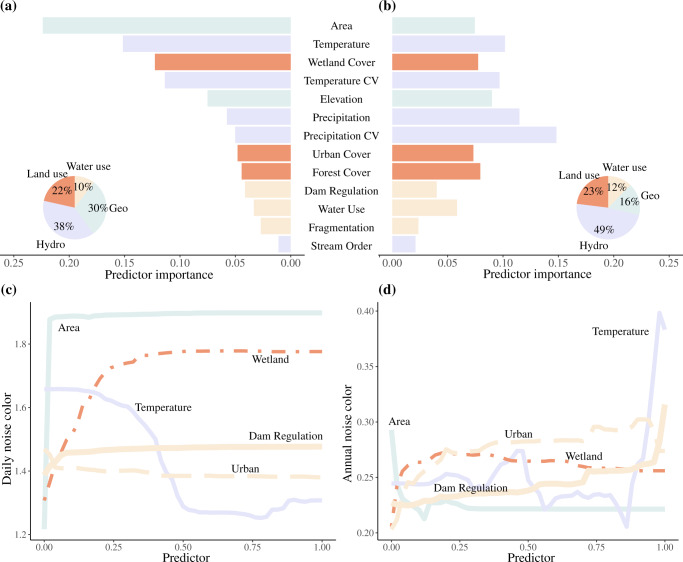
Environmental and human-related drivers of flow noise color. Relative importance of natural and anthropogenic drivers of flow noise color at the daily scale (**a**) and at the annual scale (**b**); and partial dependence plots of 5 selected drivers on daily noise color (**c**) and annual noise color (**d**). Partial dependence plots (**c**, **d**) show change in noise color attributed to each variable while removing the influence of other variables. The x-axes in **c**, **d** represent the dimensionless scales of the variables after normalizing them between 0 and 1. The color of bars in **a**, **b** indicate the different driver categories: hydroclimate (*Hydro*), geography (*Geo*), land use – land cover (*Land use*), and human activities on the water cycle (Water management, indicated as *Water use*). The relative importance of each driver was evaluated using random forest models (see Methods for model details), and is illustrated via pie charts by driver category.

Using random forest models, we then tested for associations between daily or annual flow noise color and a suite of geographic, hydroclimatic, and anthropogenic variables (land cover and water management). Random forest regression models are based on averaging multiple predictions from a multitude of decision trees, and provide a ranking of the relative importance of individual variables (or predictors) on the response variable (here, the variability of noise color; see Methods for details of the implementation of the random forest models). We found that both natural and human factors were important predictors of flow noise color across the U.S. streams and rivers (*R*^2^ = 0.72 and 0.35 for prediction of daily and annual noise color, respectively; see Figs. 3 and S6, S7, S8 and S9). Importantly, flow noise color often showed nonlinear relationships with the drivers considered, particularly upstream drainage area, wetland cover, and temperature (Fig. 3c, d). Despite this apparent complexity, some dominant drivers emerged that could explain some regionalized noise color patterns (Fig. S10). Among all drivers, upstream drainage area (geographic variable) was the single most important variable in explaining spatial variation in daily flow noise color, but its effect was comparatively weaker for annual flow color. Daily flow noise color reddened quickly across smaller to medium watershed sizes, while annual color generally showed the opposite behavior. Both daily and annual noise colors remained stable as watershed area increased further (Fig. 3c, d). Elevation was also important for both daily and annual noise color (ranked 5th out of 13 variables, Fig. 3). In turn, hydroclimate variables (e.g. precipitation, temperature) explained a total of 38% and 49% of variation in daily and annual flow noise color, respectively (Fig. 3a, b; see Table S1 for details). Variability in precipitation (Precipitation CV in Fig. 3a, b) had a stronger effect on flow noise color at annual than at daily scales, and was ranked first in terms of relative importance among all drivers considered at the annual scale. Conversely, variability in temperature (Temperature CV in Fig. 3a, b) showed a much weaker influence on annual relative to daily noise color. In addition, temperature displayed a negative association with daily noise color (*r* = −0.27, *p* < 0.01), suggesting that river flows have more random variability (at the daily scale) in relatively warmer climates. At the annual scale, flow noise color also responded strongly, and non-linearly, to temperature—showing maximum noise color values in the warmest watersheds (Fig. 3d).

Importantly, human activities (land and water uses) accounted for around a third of the spatial variation in flow noise color, both at daily and annual scales (32% and 35% in terms of total relative importance respectively, Fig. 3). Wetland coverage showed high predictive power for both daily and annual flow noise color. At the daily scale, larger wetland coverage led to reddened flow noise color, while a non-monotonic pattern (sharp increase followed by a mild decrease) was observed at the annual scale (*r* = 0.27, *p* < 0.01 for daily; *r* = 0.03, *p* < 0.01 for annual). Urbanization was also important, but showed a negative association with daily flow noise color (*r* = −0.26, *p* < 0.01). Interestingly, the overall importance of land use was quite similar at the daily and annual scales (22% at the daily scale and 23% at the annual scale, Fig. 3d). Flow management also presented a non-trivial influence on flow noise color, although not as important as that of land use (Fig. 3a, b). Specifically, the degree of regulation and the degree of fragmentation in the watershed (both controlled by dam density and size), increased both daily and annual flow noise color (*r* = 0.22 and 0.17 for regulation and *r* = 0.25 and 0.11, *p* < 0.01 for fragmentation, respectively). Dam regulation reddened daily flow noise only slightly at the daily scale, but more strongly at the annual scale—especially when approaching the upper bound of dam regulation (Fig. 3). Despite some overlap in their range of noise color values, regulated rivers showed reddened regimes relative to free-flowing ones (Fig. S11, mean of 1.68 for regulated versus 1.18 for unregulated across the gages being analyzed). This pattern was expected, as regulation and water use are known to decrease flow variability—not just its magnitude. Here we note that free-flowing and regulated rivers within each hydrologic region are subject to similar hydroclimatic conditions. Thus, differences in noise color between these two types of river ecosystems (*p* < 0.01 from a Kolmogorov-Smirnov test for regulated versus unregulated rivers) are likely driven by variation in subwatershed-level characteristics and by dam-induced alteration of their flow regimes.

### Predicting flow noise color in rivers across the CONUS

**Fig. 4:**
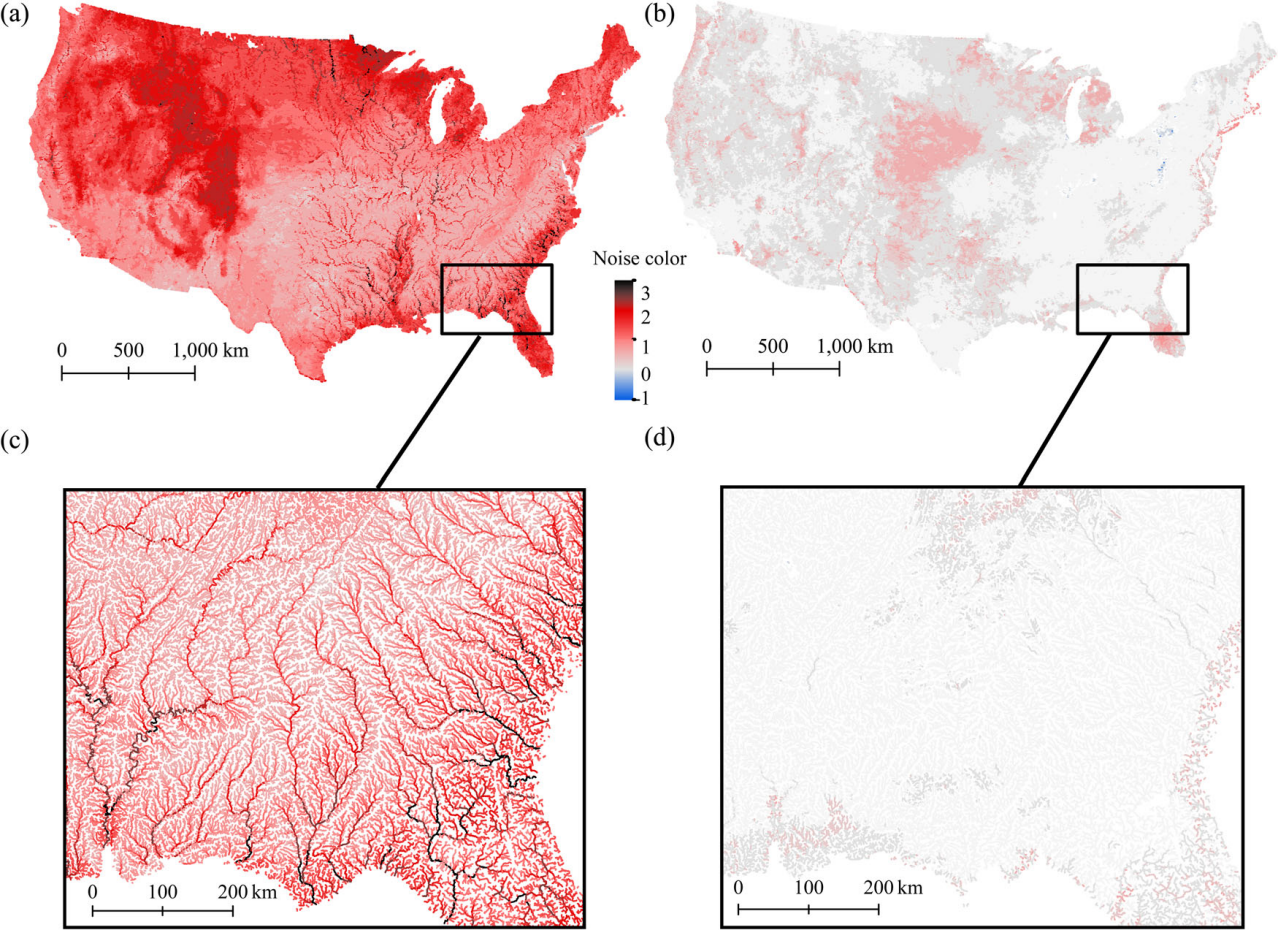
Flow noise color predicted by the random forest models at ~430,000 river reaches (~1.9 million km) across U.S. river networks. **a** Daily flow noise color; **b** Annual flow noise color. In **c**, **d** we show predicted daily and annual flow noise color for river basins in the U.S. Southeast. The contour of the CONUS credits to Copyright:© 2013 National Geographic Society, i-cubed in ArcGIS® software by Esri.

After excluding stream segments that displayed environmental conditions outside of the calibration range, we were able to predict daily and annual flow noise color for a total river length of 1,922,615 km and 1,890,951 km, respectively (Fig. 4a, b). These maps confirmed that flow noise color is highly variable across and within river networks. Daily flow noise decreased (from red to pink) from the northwest to the southeast, but increased again (from pink to black) when approaching the coastal belt (Fig. 4a). In contrast, annual flow noise color did not show any distinct spatial structure, despite some clusters of pink color in the central U.S. and Florida. Overall, we found that across the U.S. river network, daily flows showed predominantly red spectra (~53.7% of the total stream length) whereas annual flows were strongly dominated by white (random) noise colors (~93.8% of the total stream length).

When looking at finer spatial patterns within stream networks, we observed directional noise color patterns along the longitudinal gradient–particularly on daily noise color (Fig. 4c, d). For example, in the river basins of the U.S. southeast, daily noise color increased as a function of stream order and drainage area, with pink colors dominating in the headwaters, red colors in the larger main-stems, and black colors in the most downstream sections of the basin (Fig. 4c). At the annual scale, no obvious effect of network structure appeared, even when focusing on the same Southeast region (Fig. 4d).

We further compared the differences in daily noise color predictions across the CONUS between the random forest model and a spatial interpolation approach via Empirical Bayesian Kriging, EBK (see Methods for details). We found that daily noise color extracted from the spatially-interpolated grid was highly correlated with observed noise color at those gages (*r* = 0.91). In addition, daily noise color across the CONUS showed similar spatial patterns (Fig. S12a, b), with similar global means (1.21 for the random forest and 1.48 for the EBK), and more than 70.0% of the river segments were classified in the same noise color category. However, spatially-interpolated noise color via EBK does not account for the hierarchical structure of the river networks, and therefore cannot be used to conclude on the predictive ability of our models.

## Discussion

Flow noise color in rivers across the conterminous U.S. showed a larger range of values—from white to black noise for daily flows, and from blue to red for annual flows—compared to previously reported values of noise color of streamflow or air temperature and sea surface temperature from marine and terrestrial environments, which generally range from white to red^1,4^. Flow noise color captures the temporal autocorrelation structure of flow variation, and can be viewed as the watershed storage or buffering capacity of precipitation and snowmelt over time—through land surfaces, soils, aquifers, and human activities^1,28^. Consequently, streamflow is expected to display stronger temporal correlation (pink to black color as found here) than precipitation, which generally shows uncorrelated or short memory behavior (especially at daily scales)^29–31^. Flow noise color showed large spatial heterogeneity and inconsistencies at different temporal scales—daily to seasonal, and even across years, consistent with previous research^1,32^. However, unlike nearly uniformly white annual flow noise color reported by other studies^1^, we found that more than 20% of the gages showed non-white color, indicating that water storage variation significantly deviates from zero across years. The large variability of flow noise color across river networks further shows that looking for regionalized flow patterns without detailed information on the river network structure (e.g., topology, degree of regulation) may be misleading, as influences on flow variation have cumulative effects along the river network. Overall, our results demonstrate that the structure and drivers of noise color in river networks may fundamentally differ from those previously reported from marine and terrestrial ecosystems, and highlight the importance of human influences driving variation in river flow regimes at local to continental scales.

Our results highlight the impacts of anthropogenic hydrologic alteration on patterns of temporal variability in streamflow, and further confirm the complexity of streamflow dynamics, which integrates signatures of the hydrosphere and anthroposphere across spatiotemporal scales^33,34^. We found that drivers such as upstream drainage area, topography, precipitation, and temperature all exhibited strong correlation with flow noise color. In agreement with previous work^32,35–37^, drainage area was the variable that correlated the most with temporal persistence of daily flow variation. The scaling of flow variability with basin area is likely driven by heterogeneity in watershed runoff transport processes^38^. For instance, flow regimes that would be flashier in smaller (sub)watersheds can become smoother when integrated across larger drainage areas. Climate non-stationarity is critical for flow variability, as we found that temporal variability in precipitation is strongly correlated with flow noise color, especially at the annual scale. The observed temperature effects are also likely linked to precipitation-driven versus snowmelt-driven hydrographs. Snow influence is less important in rivers located in warmer basins; thus, antecedent conditions may not contribute much to flow discharge persistence in those areas. In turn, cooler basins tend to have more snow and longer memories^39^, leading to the observed reddened spectra of rivers in these regions.

Importantly, we found that human-related factors (land use and flow management) strongly correlated with spatial variation in flow noise color, even if not as important as natural drivers. Flow regimes in urbanized landscapes often have large flow variability and peak magnitude at the daily scale, as infiltration is limited, and water evacuates watersheds faster via runoff, often in the form of noisy (white), pulsated flows (a symptom of the “urban stream syndrome”^22^). In contrast, wetlands act as natural “sponges” by storing water, regulating quickflow and baseflow, increasing infiltration and decreasing surface runoff^40^. It is thus not surprising that watersheds and regions with higher relative wetland cover generally present smoother flows and thus reddened spectra. As natural land cover is increasingly altered due to societal development, our results underscore the importance of human-driven hydrologic alteration (via damming and alteration of natural land covers) in shaping patterns of flow variation across large spatial scales.

Flow regulation by dams is a major factor affecting river connectivity globally^19^. In the U.S. alone, dams affect nearly 1 million km of rivers^41^, disrupting many physical and ecological processes^26,42,43^. Dams tend to stabilize flow variability by muting high flows, increasing base flows, and reducing the frequency of high and low-flow events through reservoir operations^16^. Consequently, regulation tends to smooth flow variability—and homogenize flow regimes across large spatial scales^17^. However, we note here that flow regulation was not universally associated with “reddened” flows, which could be potentially caused by variation in reservoir management goals^16^. As temporal autocorrelation in the environment may change substantially over time^44^, flow regimes from regulated and non-regulated rivers may diverge from each other under increasingly variable hydroclimates^23^. Because river ecosystem structure and functioning is sensitive to the timing, frequency, and magnitude of environmental extremes^45–47^, noisier river flows may likely lead to declines in ecosystem stability^7^. This may be particularly true in small systems (e.g., in headwaters), where small changes in flow regime could turn perennial into intermittent systems—leading to a “blueing” of their flow regimes and a shift in their ecosystem structure and services^48,49^.

Our results have broad implications for conservation and management of riverine ecosystems and flow-dependent biodiversity. Most research on regional patterns of streamflow alteration, and widely-used metrics aimed at capturing hydrologic alteration (e.g., the Indicators of Hydrologic Alteration, IHA), have largely focused on the time domain (e.g.,^23,45–47^). We propose that these indices could be complemented with spectral-based metrics that result from partitioning signal and noise in the frequency domain, like those investigated here and in previous similar research (e.g.,^1^). Flow noise color is an example of a facet of flow variation that may not be captured via traditional time-series analyses in the time domain—in fact, two flow regimes with similar levels of variation could fundamentally differ in their temporal autocorrelation structures (e.g., a flood-control versus a hydropeaking reservoir). Additionally, changes in the color of environmental noise of other regimes, such as light^50^ or sediment regimes^51^, have not been investigated to the same extent than flow in river ecosystems–but they could all control key processes like ecosystem productivity. We contend that anthropogenic change to watersheds, via increases in imperviousness (due to urbanization) and in water residence time (due to dam regulation) may be affecting riverine biodiversity persistence, among other mechanisms, by changing the frequency and predictability of extreme events^1,52^. For instance, the reddening of flow regimes due to dam regulation may increase extinction risk by lengthening the time spent in unfavorable environmental conditions, reducing demographic rescue (though different life history strategies or life stages are expected to respond differently^53^), while also promoting non-native species establishment^17^. Investigating the link between changes in flow seasonality or stochasticity and ecological outcomes remains an important research frontier for implementation of environmental flows (see^13,48,49,54^).

The patterns we revealed also have key ecological implications in the context of ongoing and predicted climate change. Our results suggest that any changes in the mean and variance of temperature and precipitation—which both have been predicted^55^—will have consequences for flow noise color in the future. For instance, widespread increases in mean surface temperature may lead to changes in the volume and timing of snowmelt runoff^56^, thereby decreasing the seasonal persistence of flow regimes^39^. The reddening and spatial homogenization of temperature regimes predicted globally^57,58^ may also participate in the large-scale convergence of historically distinct flow regimes and their associated floras and faunas. Leveraging long-term high-resolution discharge data such as done in this study could allow us to explore the concomitant effects of changes in spatial and temporal autocorrelation of climatic variables that have occurred over the last decades with the effects of river regulation on flow noise color. Unveiling the full range of impacts that climate non-stationarity and human management may have on streamflow regimes is a necessary first step to anticipate, and potentially mitigate further river ecosystem degradation.

## Methods

### Selection of Streamflow Gages

The streamflow datasets used in this study are daily streamflow time series from the United States Geological Survey (USGS) flow gages (access at: https://waterdata.usgs.gov/nwis/). We used daily records from 7504 gages and annual records from 2594 gages. Gages were selected based on the following criteria: (1) Recording period of at least 15 consecutive years, within 1960 to 2019; (2) missing records being less than 5% of the total length. Any missing data was estimated by linear interpolation. When provided, sub-daily flow values were converted to mean daily discharge. To illustrate the impact of temporal scales on noise color, annual flow noise color was further estimated based on the subset of 2594 streamflow time series that had 50 years of complete daily records.

### Flow noise color

The color of environmental noise from the streamflow time series can be determined through linear regression of the power spectrum density and the characteristic frequency of streamflow. We removed seasonal components (annual cycles) and long-term trends (long-term linear trend) of the flow discharge (by using the “stl” function, “Seasonal Decomposition of Time Series by Loess”, in the stats package in R; see Cleveland, et al. ^59^. for details of the decomposition) to obtain the remaining residuals of the time series. Then we calculated the frequency spectrum of the residuals for each flow time series. The power spectrum density *P*_*k*_ can be estimated by the Fast Fourier Transform (FFT) as follows:

1 $${F}_{k}={T}^{-0.5}\mathop{\sum }\limits_{t=0}^{T-1}\,{f}_{t}{e}^{i2\pi {tk}/T},\, k=0,\, 1,\cdots,\, T-1$$

2 $${P}_{k}={F}_{k}^{2}$$

where *T* is the Nyquist frequency; *t* is the current record; *k* is the current frequency; *F*_*k*_ is the amount of frequency *k/T* in the streamflow signal; *i* is $\sqrt{-1}$; *f*_*t*_ is the detrended streamflow records. Then, noise color can be determined as follows:

3 $$\log {P}_{k}=-{alog}\left(\frac{k}{T}\right)+b$$

where *a* is the coefficient representing noise color; *b* is the intercept of the regression line. To account for the range of variability in noise colors, in this study we followed a similar classification to that used in Sabo and Post^1^ and Vasseur and Yodzis^4^. We classified noise color as follows: blue noise (−1.5 ≤ *a* ≤ −0.5), white noise (−0.5 ≤ *a* ≤ 0.5), pink noise (0.5 < *a* ≤ 1.5), red noise (1.5 < *a* ≤ 2.5), and black noise (*a* > 2.5).

We explored the effects of detrending the data in different ways: 1) By removing the long-term linear trend only; 2) By removing both the long-term linear trend and periodic (mostly annual) cycles; and 3) By using the raw data. Noise color estimates remained virtually unchanged regardless of the detrending procedure chosen (Fig. S1). We show results on detrended and deseasonalized data for consistency with previous work that calculates noise color based on “residuals” (e.g., as reported in Sabo and Post^1^ and Vasseur and Yodzis^4^).

### Random Forest Model

We used random forest models to identify the main drivers of flow noise color at daily and annual scales, and applied the models to predict noise color for each reach of the river network across the CONUS. We developed the following workflow:

1. Explanatory variables. We selected 13 variables from 4 general categories of drivers: geography, hydroclimate, land use land cover, and water management. See Table S1 for details on the variables. Basin characteristics, such as drainage area and elevation, were extracted from Falcone^60^ and Lehner and Grill^61^. Long-term mean annual precipitation and temperature estimates at these gages, between 1964–2017, were extracted from the 4-km monthly PRISM datasets^62^, and climate characteristics at each gage were estimated by bilinear interpolation. The land use/land cover data, including forest, wetland, and urbanization estimates were derived from Kroeker, et al. ^5^. for each corresponding watershed (hydrologic unit code level-10, HUC10). Metrics from 2006 were used to represent overall conditions. Anthropogenic impacts, namely degree of regulation (DOR) and degree of fragmentation (DOF), were extracted from Grill, et al. ^19^. and Lehner and Grill^61^ for each stream segment. Human water use in 2005 was used to represent the overall mean annual freshwater withdrawal^63^ from 1985–2010, as estimated by the sum of water uses across HUC10.
2. Random forest implementation. The random forest models were fitted using a 10-fold cross-validation, and were trained on 80% of the gages (randomly selected) and tested on the remaining 20% of the gages. Details of the random forest algorithm can be found in Breiman^64^. The implementation of the random forest models was done with the R package randomForest^65^. The out-of-bag error (mean squared error, MSE) was used to evaluate the model performance. The random forest model was first used to describe the relative importance of each variable in explaining spatial variation in flow noise color (for details on the estimation of relative importance, see Breiman^64^). The same procedures were used to construct a random forest model with annual flow noise color. For daily flow noise color, the final model had an out-of-bag error of 0.12 on the training dataset. On the testing dataset, the Pearson’s correlation coefficient (*r*) was 0.85, percentage bias was 1.2% and MSE was 0.11. In comparison, for annual flow noise color, the final model had a MSE of 0.09, and *r* was 0.57.
  Most of the prediction error (i.e., the difference between predicted and observed daily flow noise color) fell within the range of −0.25 to 0.25 units (mean = 0.0007; percentage bias = −0.1%). Misfit maps for training and calibration datasets are shown separately in Fig. S6a, b. Prediction errors did not vary systematically across hydrologic regions (HUC2) or stream orders (Fig. S7), for neither the training nor the testing data sets. Overall, over three quarters of the gages (76% of the gages in the calibration data set, and 75% of the gages in the validation data set) were correctly assigned to their noise color category.
3. Mapping flow noise color. Finally, we used the calibrated models to predict daily and annual flow noise color for each individual river reach across the CONUS (from Grill, et al. ^19^. and Lehner and Grill^61^). Noise color at the midpoint of each river reach was selected to represent the overall color of the reach (the average length of the river reach is about 4 km). The 13 aforementioned predictors at these points were obtained in the same manner as those used for the 7504 gages. River reaches were selected only if the associated attributes were within the range of the corresponding predictor variables observed among the gages used to calibrate the models (i.e. 7504 and 2594 gages for daily and annual noise color, respectively). This step allowed us to avoid extrapolating flow noise color to unobserved environmental conditions. Based on the availability of environmental and anthropogenic data, we were able to predict flow noise color for a total of 437,766 stream segments, corresponding to 1,922,615 km and 1,890,951 km of the U.S. hydrographic network for daily and annual noise color, respectively.
4. Evaluation of global prediction of flow noise color. We compared flow noise color estimates from the random forest to those obtained by spatial interpolation via Empirical Bayesian Kriging (EBK). We ran EBK in ArcGIS 10.7, with optimal interpolation parameters (covariance and regression coefficients) and accounting for error by estimating the semivariogram (for details of the EBK, see Gribov and Krivoruchko^66^). We mapped observed noise color onto a ~2 × 2 km grid across the CONUS, and extracted noise color values at the mid-point of each flow line from the gridded map.

### Reporting summary

Further information on research design is available in the Nature Portfolio Reporting Summary linked to this article.

## Supplementary information

Supplementary information

Reporting Summary

## Data Availability

The datasets generated in this study are available at the 10.6084/m9.figshare.21428061.The streamflow datasets used in this study were retrieved from the United States Geological Survey (USGS) flow gages (access at: https://waterdata.usgs.gov/nwis/). The precipitation and temperature datasets were retrieved from PRISM Climate Group at https://prism.oregonstate.edu. The water management datasets were retrieved from USGS Data Release at 10.5066/F7XW4J1J. The gage properties were retrieved from USGS at 10.3133/70046617. The datasets of upstream catchment area and stream order were retrieved at https://figshare.com/articles/dataset/Mapping_the_world_s_free-flowing_rivers_data_set_and_technical_documentation/7688801.

## Code availability

The code of the analyses performed in R (version 4.0.5) can be accessed at 10.5281/zenodo.7585325.
