# Supplementary material for: The color of environmental noise in river networks
Source: Nat Commun. 2023 Mar 28;14:1728. doi: 10.1038/s41467-023-37062-2 (PMC10050181; doi:10.1038/s41467-023-37062-2)
Supplement: Supplementary file 1 — Supplementary information [file 41467_2023_37062_MOESM1_ESM.pdf]

Supplementary information for:

The color of environmental noise in river networks

Tongbi Tu[1,2,*], Lise Comte[3], Albert Ruhi[2]

[1] School of Civil Engineering, Sun Yat-sen University, Guangdong, 519082, China

[2] Department of Environmental Science, Policy & Management, University of California, Berkeley, Berkeley CA 94702, USA

[3] School of Biological Sciences, Illinois State University, Normal, IL 61790, USA

**** Corresponding author:*** *Tongbi Tu, tutb@mail.sysu.edu.cn*

This file contains:

Supplementary Fig. 1. Comparison of daily flow noise color estimates obtained under different detrending schemes.

Supplementary Fig. 2. Temporal scaling regimes of the spectral density of daily flows at the four selected gages.

Supplementary Fig. 3. Comparison of noise color estimates for daily flow data on 7,504 gages, using all timescales (“global fit”) vs. a narrower window (“local” fits).

Supplementary Fig. 4. Geographic variation in flow noise color at the daily scale.

Supplementary Fig. 5. Geographic variation in flow noise color at the annual scale.

Supplementary Fig. 6. Spatialization of prediction errors at the gage-level.

Supplementary Fig. 7. Spatialization of prediction errors at the regional scale.

Supplementary Fig. 8. Boxplots of daily noise color across the CONUS.

Supplementary Fig. 9. Boxplots of annual noise color across the CONUS.

Supplementary Fig. 10. Drivers of noise color within river networks across the CONUS, and predicted daily flow noise color using random forest.

Supplementary Fig. 11. Daily flow noise color under different degrees of regulation.

Supplementary Fig. 12. Comparison of predicted daily noise color across stream segments in the CONUS.

Supplementary Table 1. Description of the geographic, hydroclimatic, and anthropogenic natural and human-related variables considered in the random forest models, with associated spatial scales and data sources.

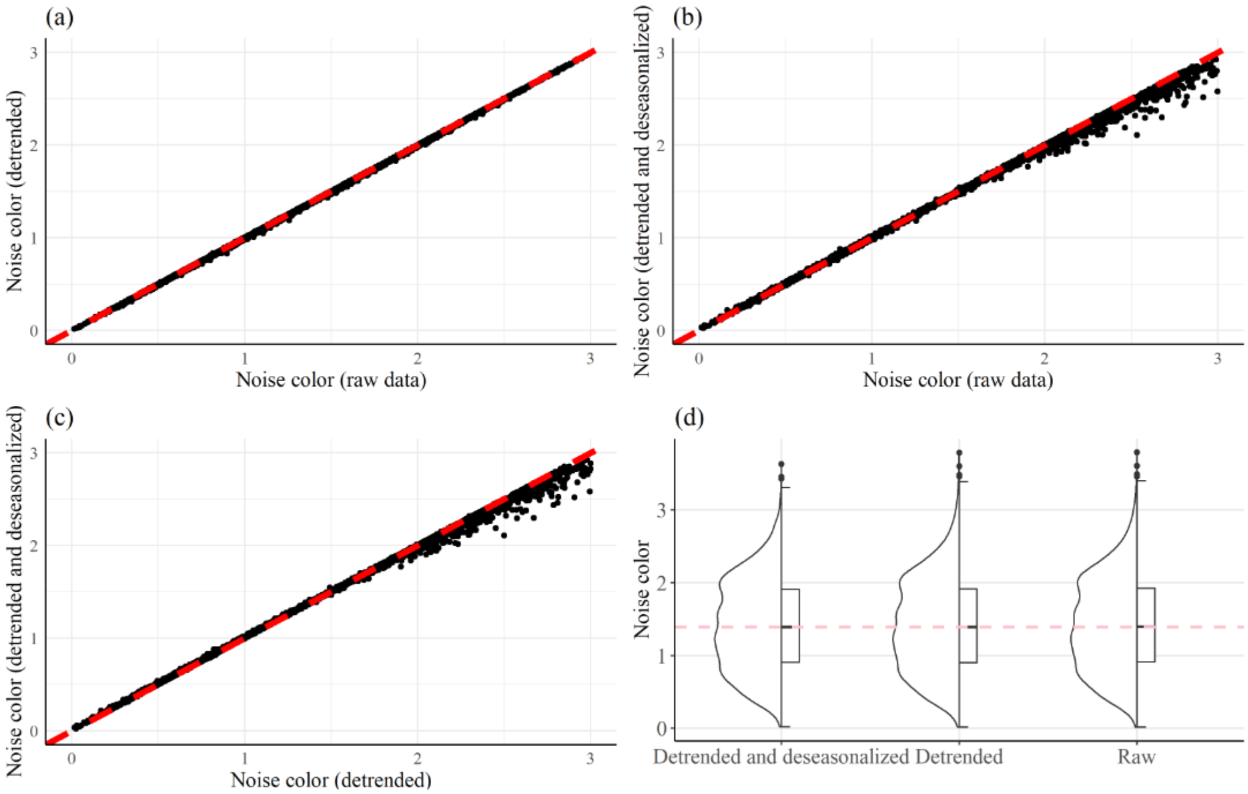

Fig. S1. Comparison of daily flow noise color estimates obtained under different detrending schemes: no detrending ("raw data", i.e., spectral analyses based on observed flows); removing the long-term linear trend only ("detrended"); and removing both the long-term linear trend and periodicity in the data ("detrended and deseasonalized"). We note that patterns were almost identical regardless of the option chosen, with correlation coefficients for each of these pairs ranging from 0.999 to 1.00. In (a), (b), and (c), pairwise comparisons are shown against a 1:1 line, with each dot being a streamgage record (N=7,504). In (d), we show the distribution of daily flow noise color values based on raw (for observed), detrended, and detrended and deseasonalized flows. Right-half box-plots show almost identical median and interquartile ranges across metrics.

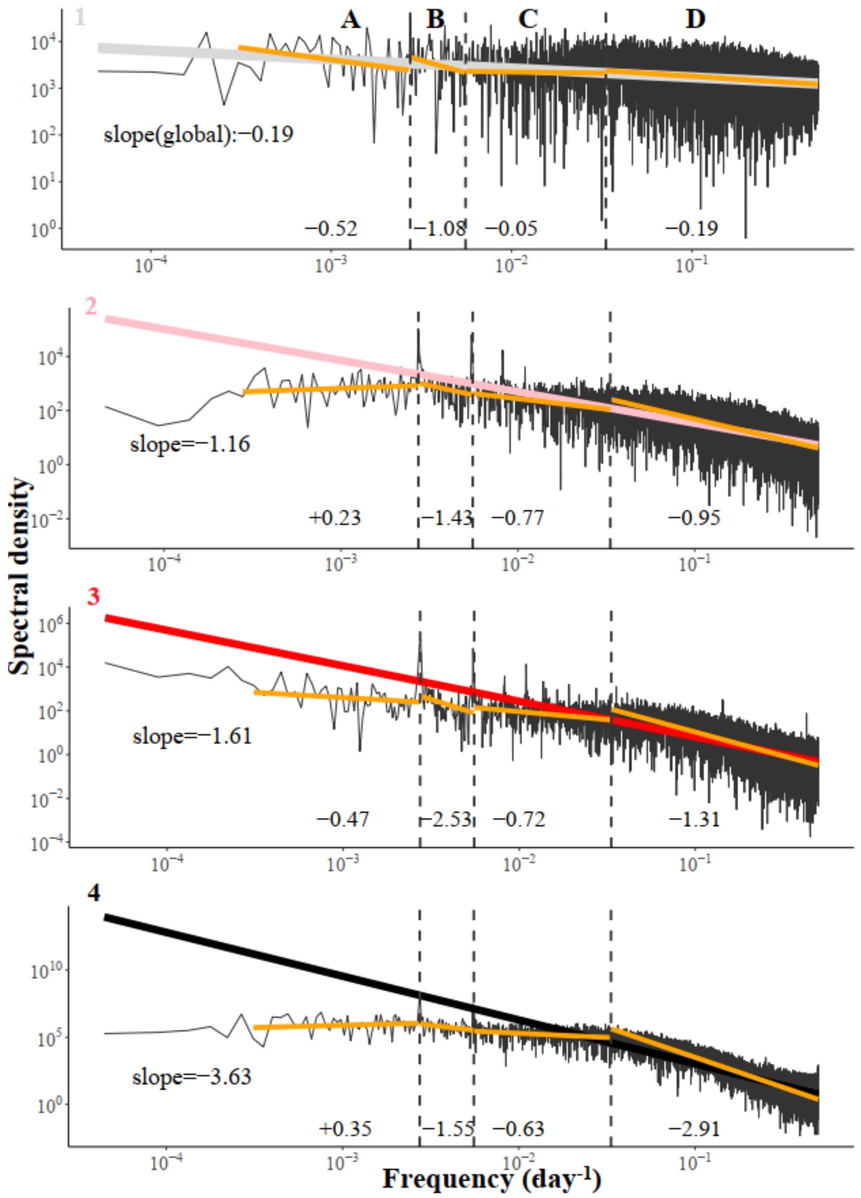

Fig. S2: Support for multiple scaling regimes when computing spectral density based on daily flows at the four selected gages. The solid lines over the entire frequency range indicate the "global" regression line when considering all timescales (as in Fig. 2). In turn, the piece-wise "local" regression lines illustrate different scaling regimes at (A) 1-10 years, (B) 0.5 to 1 year, (C) 30 to 180 days, and (D) 7 to 30 days. Values within each bin indicate the slope of each "local" regression, which tended to flatten as temporal scales increased (i.e., from D to A). See Fig. S3 for similar analyses on the whole set of 7,504 gages across the U.S.

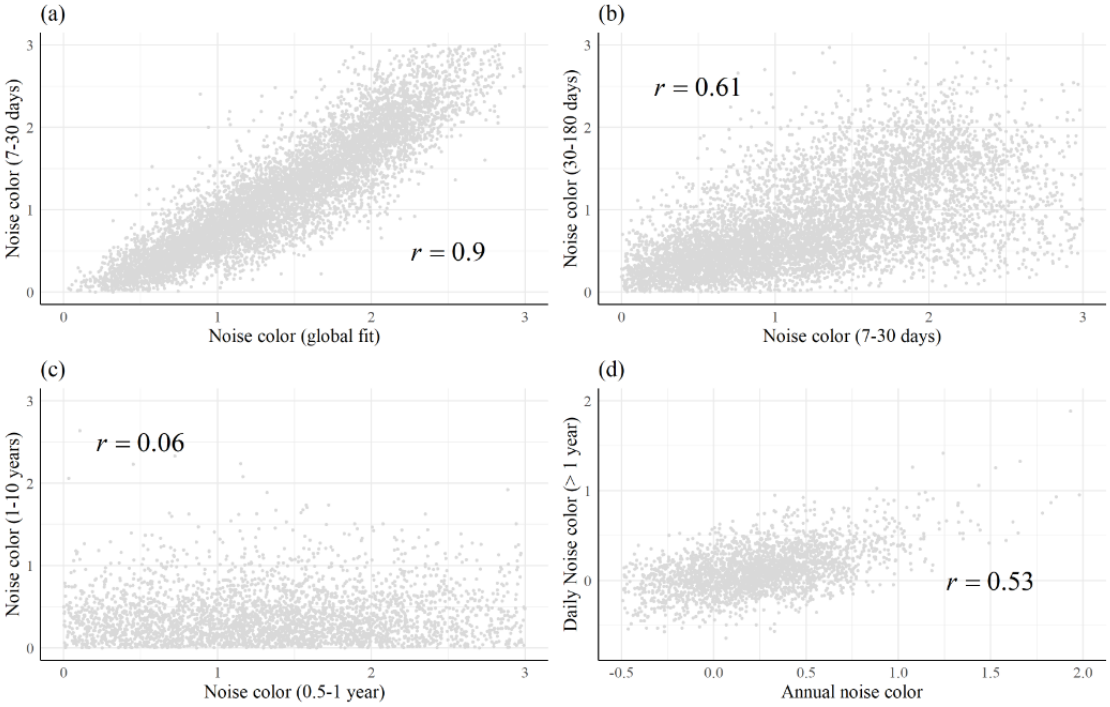

Fig. S3. Comparison of noise color estimates for daily flow data on 7,504 gages, using all timescales ("global fit") vs. a narrower window ("local" scaling regimes). (a) Comparison of noise color estimates obtained over 7 to 30-day frequencies vs. estimates using the "global fit"; (b) Comparison of noise color estimates obtained over 30 to 180-day frequencies vs. 7-30 day frequencies; (c) Comparison of noise color estimates obtained over a 1 to 10-year frequencies vs. 0.5 to 1-year frequencies; and (d) Comparison of daily noise color estimates obtained over frequencies larger than 1-year vs. annual noise color estimated by using the corresponding annual mean flow records. Noise color values were extracted from the slopes of the linear regressions of the spectra against its frequency (see examples in Fig. S2). Each dot is one streamgage record (N=7,504). Pearson correlation coefficients (r) indicate the strength of linear associations between color noise values in each case. We note that noise color generally whitened (decreased noise color values) as timescales increased from sub-monthly to annual scales, and noise color using the global fit was strongly correlated with values at the sub-monthly scale (r=0.9).

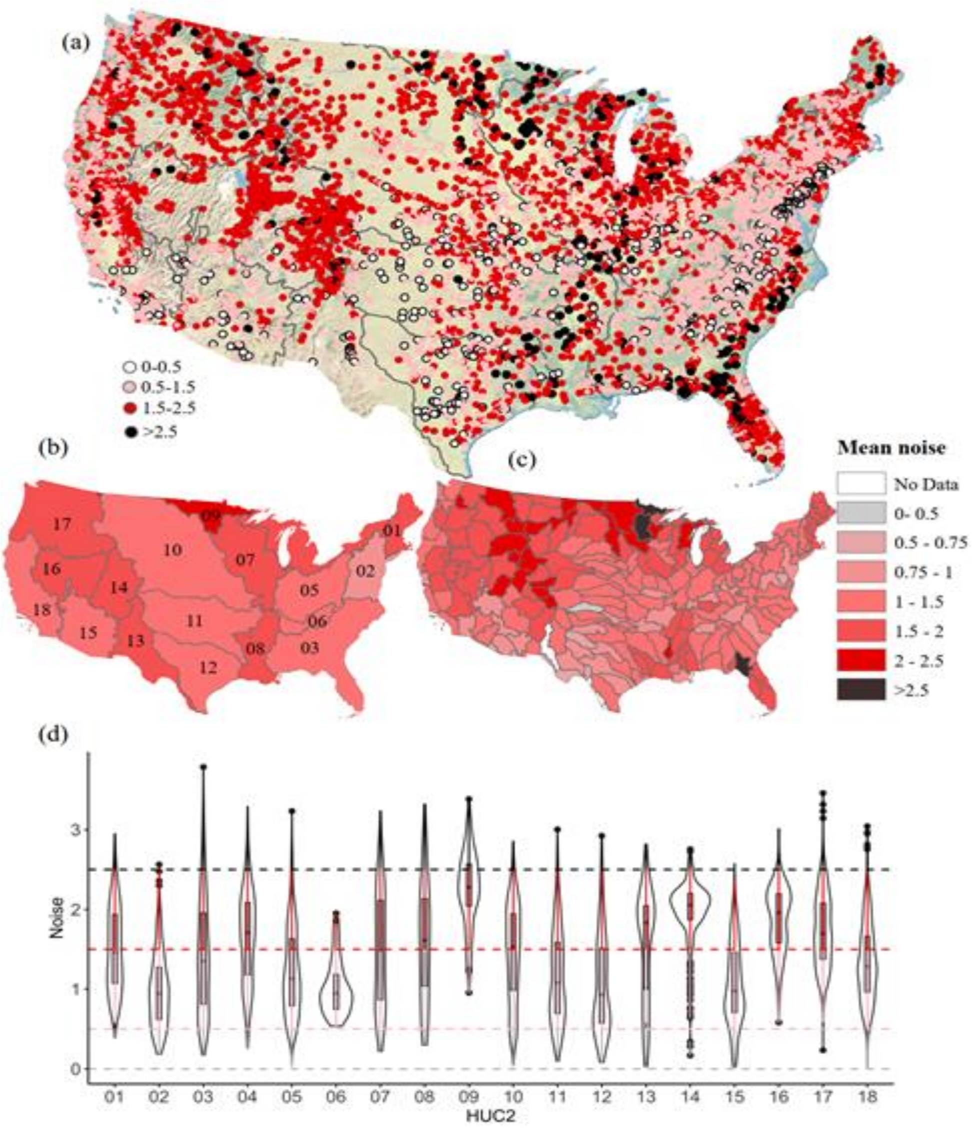

Fig. S4. Geographic variation in flow noise color at the daily scale. (a) Map of noise color patterns based on long-term mean daily flows. Each dot indicates one of the 7,504 USGS stream gages considered in this analysis. Flow noise was classified into 4 colors based on the flow noise color coefficient, i.e., white noise (-0.5-0.5); pink noise (0.5-1.5); red noise (1.5-2.5) and black noise (>2.5). The grey lines indicate hydrologic region boundaries i.e., Hydrologic Unit Code level-2 (or HUC2); (b) Mean noise color in each of the hydrologic regions (HUC2); (c) Mean noise color in each of the Hydrologic Unit Code level-4 (HUC4), which are nested within hydrologic regions; (d) Violin plots showing the distribution and interquartile range of noise color distribution by HUC2. The different horizontal dashed lines indicate the thresholds across noise color bins (no negative values were recorded). The background of the CONUS credits to Copyright:© 2013 National Geographic Society, i-cubed in ArcGIS® software by Esri.

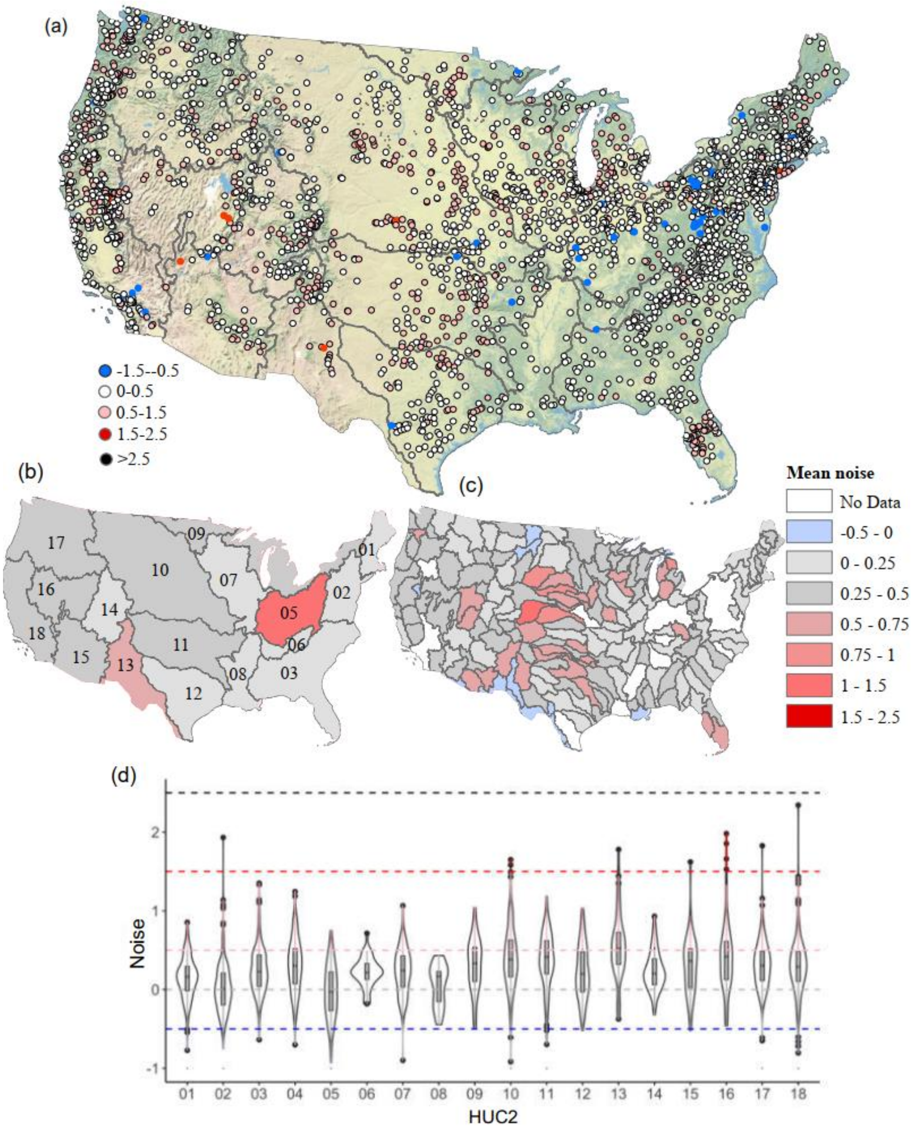

Fig. S5. Geographic variation in flow noise color at the annual scale. (a) Map of noise color patterns based on long-term mean annual flows. Each dot is one of the 2,597 USGS stream gages considered in this analysis. Flow noise was classified into 4 colors based on the flow noise color coefficient, i.e., blue noise (-1.5-0.5); white noise (-0.5-0.5); pink noise (0.5-1.5); red noise (1.5-2.5), and black noise (>2.5). Grey lines indicate hydrologic region boundaries, i.e., Hydrologic Unit Code level-2 (or HUC2); (b) mean noise color in each of the hydrologic regions (HUC2); (c) mean noise color by Hydrologic Unit Code level-4 (HUC4), which are nested within hydrologic regions; and (d) Violin plots showing the distribution and interquartile range of noise color distribution by HUC2. The different horizontal dashed lines indicate the thresholds across noise color bins. The background of the CONUS credits to Copyright:© 2013 National Geographic Society, i-cubed in ArcGIS® software by Esri.

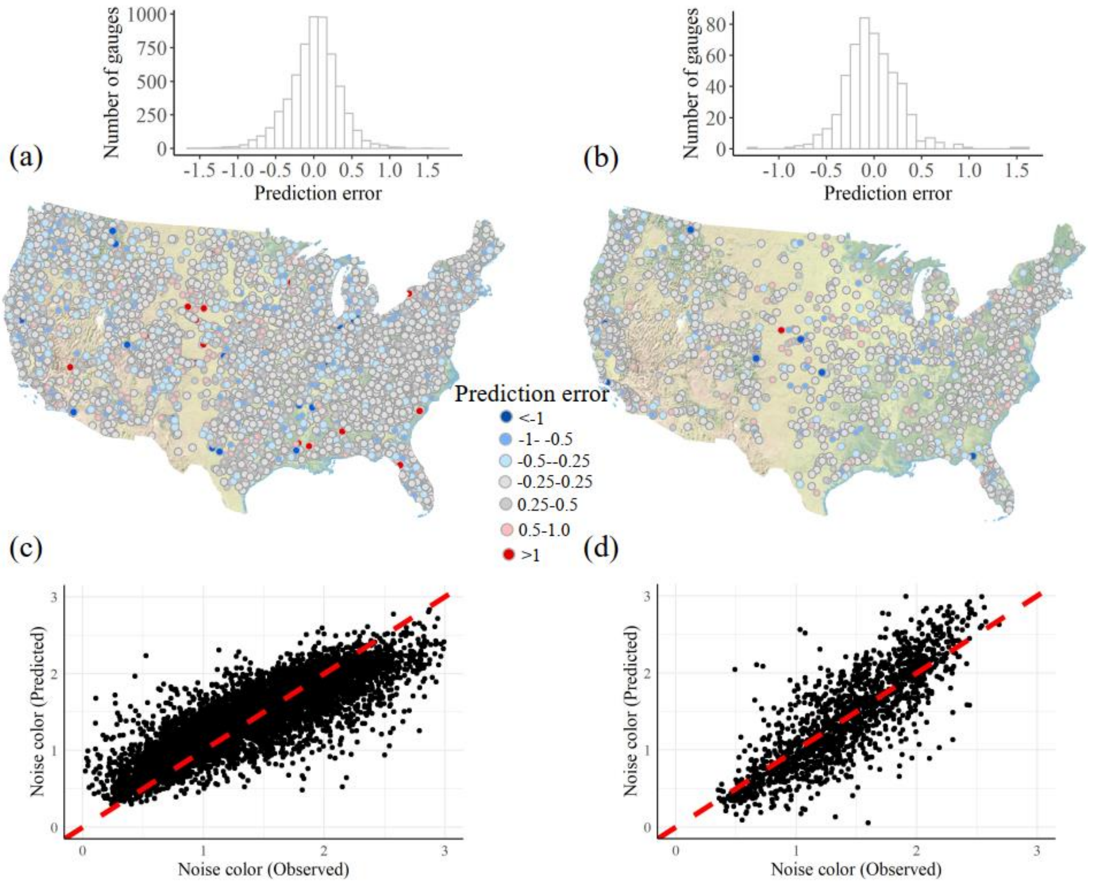

Fig. S6. Spatialization of prediction errors at the gage level. We show prediction errors (i.e. differences between predicted and observed values) of the random forest models predicting daily flow noise color for the (a) training and (b) testing datasets; and comparisons between predicted and observed noise color for the (c) training and (d) testing datasets. The histograms in (a) and (b) show the overall distribution of prediction errors, and the red dotted line in (c) and (d) is the 1:1 line, each dot being a streamgage record (N=7504). 76% of the training (calibration) gages and 75% of the testing (validation) gages were correctly assigned to their noise color category. The background of the CONUS credits to Copyright:© 2013 National Geographic Society, i-cubed in ArcGIS® software by Esri.

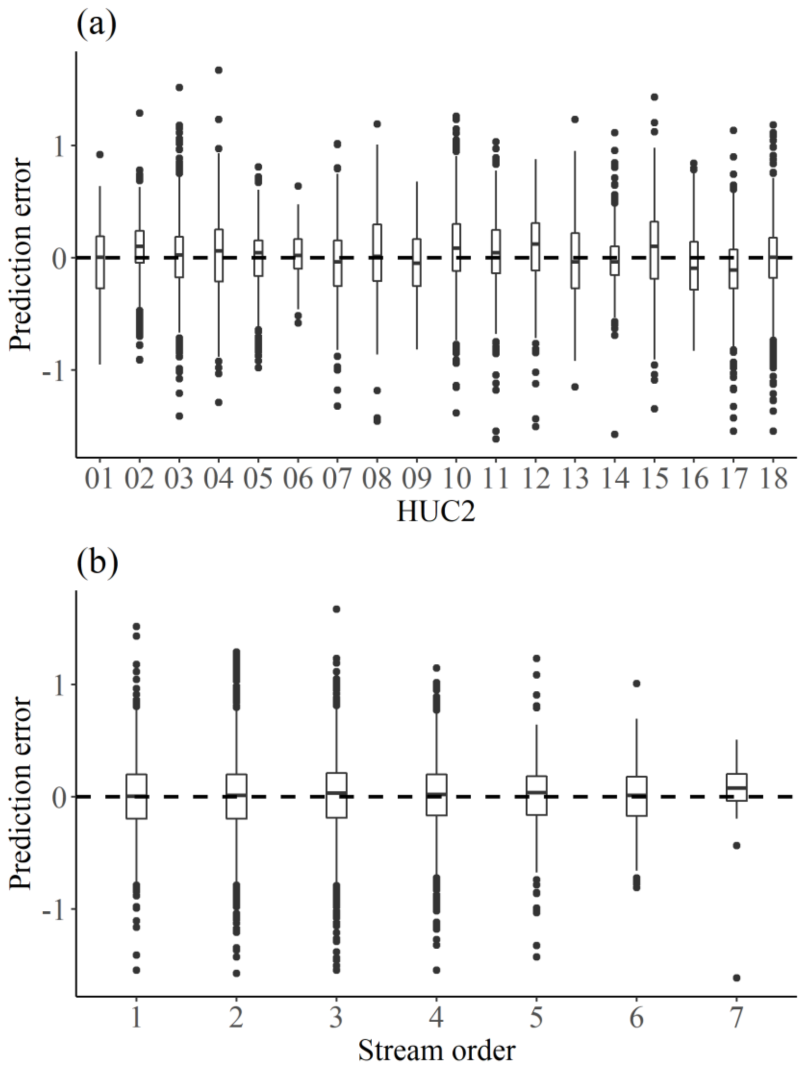

Fig. S7. Spatialization of prediction errors at the regional scale. Range of variability of prediction errors by hydrologic region (HUC2). Positive values denote overestimated noise color (i.e., prediction is 'redder' than reality, while negative values denote underestimated noise color (i.e., prediction is 'whiter' than reality). Linear models also indicated no systematic variation in prediction errors by stream order ($F_{7,\,5993}$= 0.4318, p = 0.883 for training data set; $F_{7,\,1493}$= 1.037, p = 0.403 for testing data set). We also found there are only very small effects when assessing the influence of HUC2 (η^2 = 0.02, "medium" effects are considered to start at 0.06), and negligible effects when assessing the influence of stream order ($\eta^2 = 1.04\times10^{-3}$). See Fig. S6 for gage-specific patterns in prediction errors.

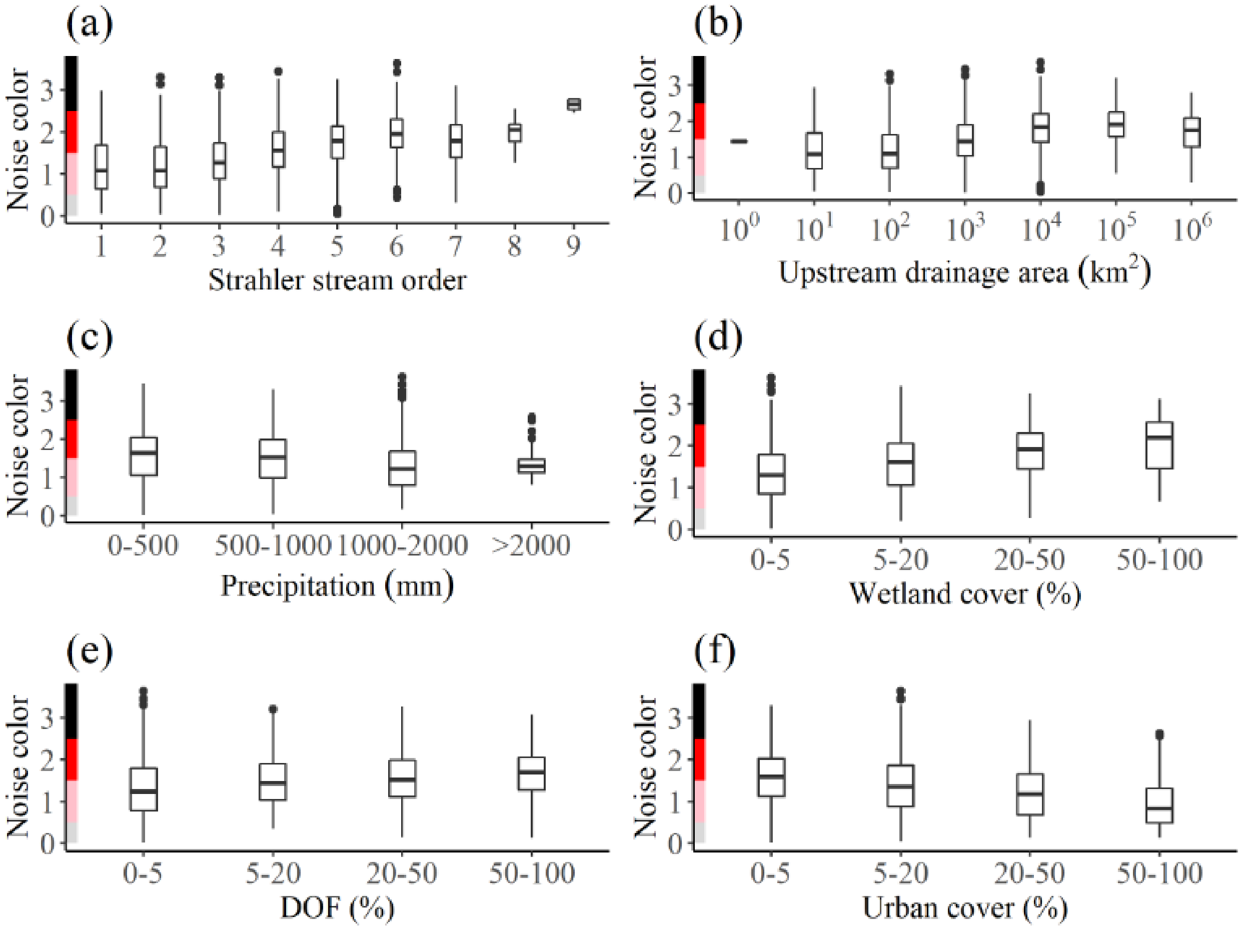

Fig. S8. Boxplots of daily noise color estimates across the CONUS according to Strahler stream order (a); upstream drainage area (b); precipitation (c); wetland cover (d); degree of fragmentation (e) and urban cover (f).

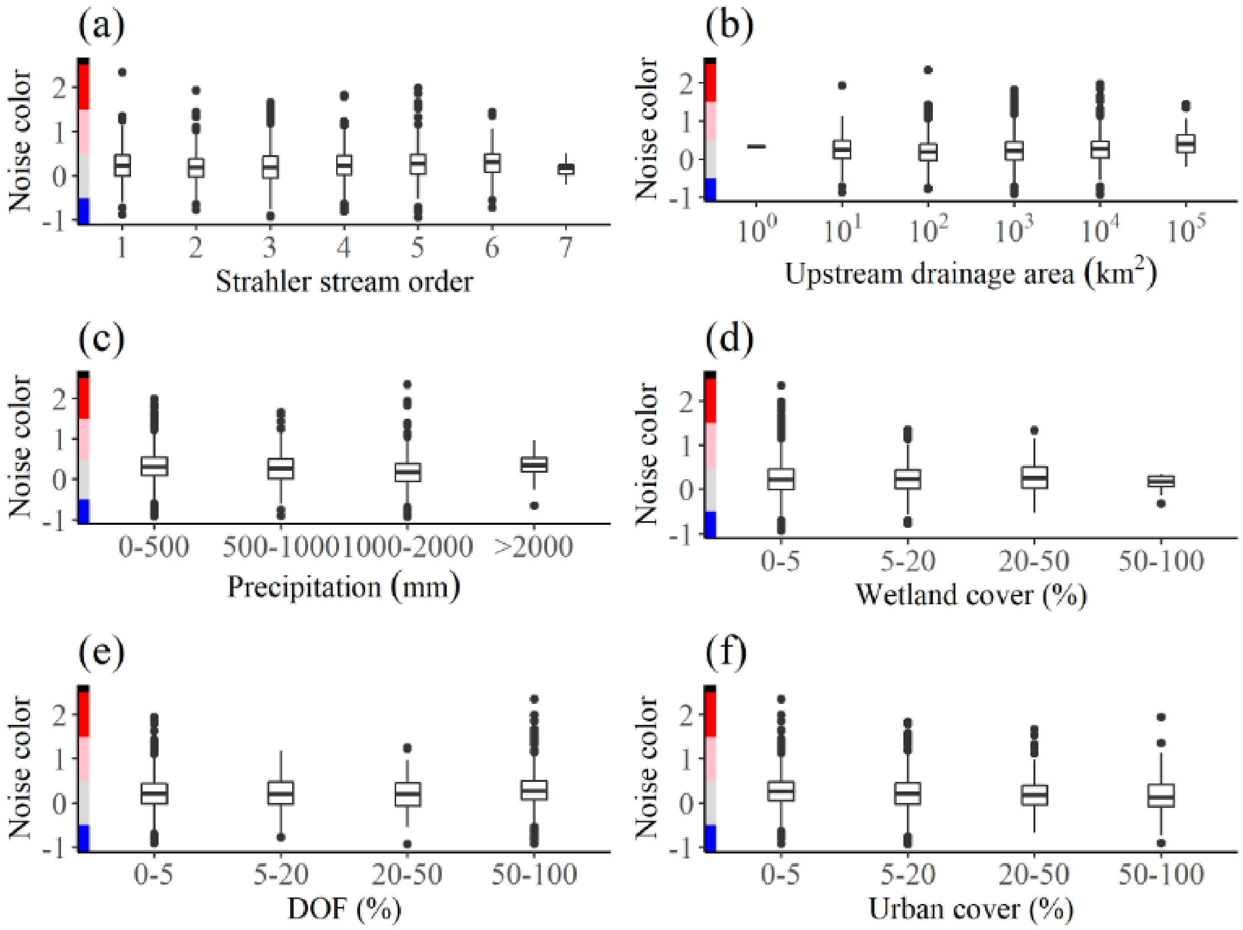

Fig. S9. Boxplots of annual noise color across the CONUS according to Strahler stream order (a); upstream drainage area (b); precipitation (c); wetland cover (d); degree of fragmentation (e) and urban cover (f). The colors on the vertical axis in each subplot indicate the range

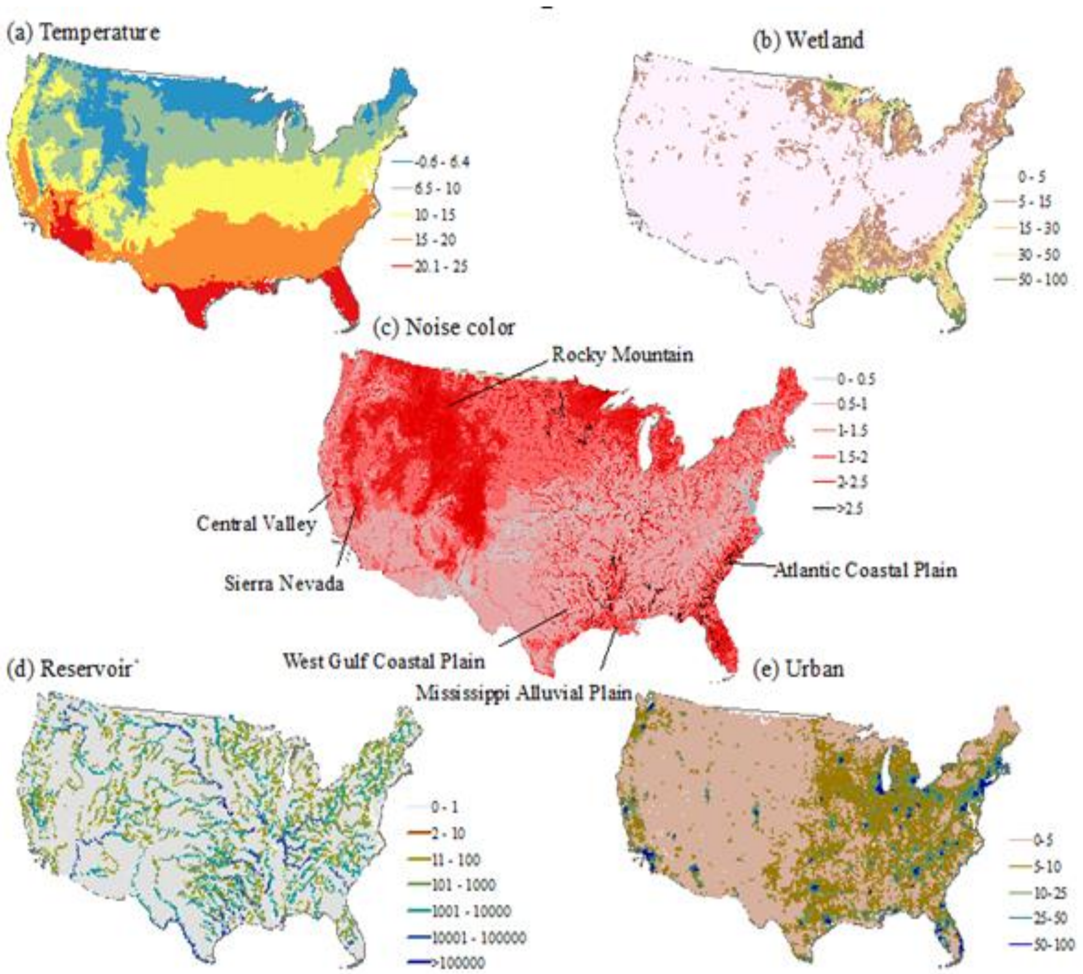

Fig. S10. Drivers of noise color within river networks across the CONUS, and predicted daily flow noise color using random forest: (a) Long-term mean annual temperature for each stream segment; (b) Wetland coverage (in %) at the sub-watershed level (HUC 10); (c) Daily flow noise color predicted using random forest for each stream segment across the CONUS (see Methods for details); (d) Upstream cumulative reservoir volume for each stream segment; (e) Degree of urbanization (in %) in the watershed (HUC 10) associated with each stream segment. The background of the CONUS credits to Copyright:© 2013 National Geographic Society, i-cubed in ArcGIS® software by Esri.

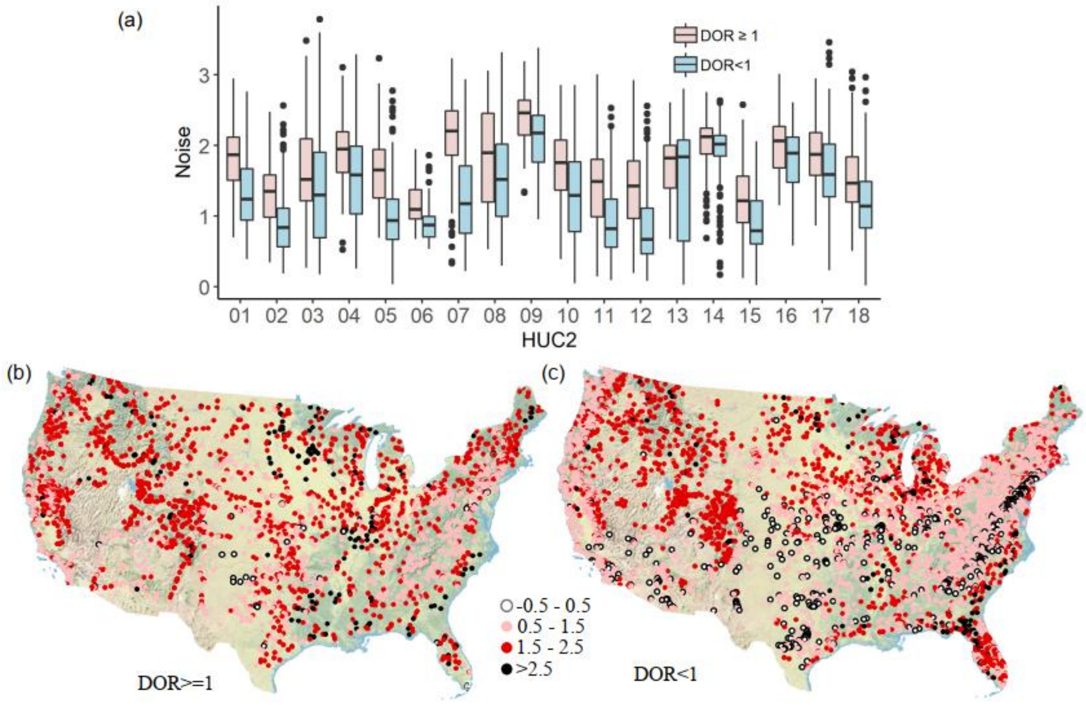

Fig. S11. Daily flow noise color under different degrees of regulation (DOR).

(a) Distribution of daily flow noise color at each region at the HUC2 level across the CONUS; (b & c) Maps of observed flow noise color for gages located in river reaches with DOR≥1 (b) *vs*. DOR<1 (c). DOR (between 0-100) quantifies the impact of dam storage (flow regulation) on altering downstream natural flow regimes, and DOR=1 here refers to no or minimal impact of flow regulation at the river reach. The background of the CONUS credits to Copyright:© 2013 National Geographic Society, i-cubed in ArcGIS® software by Esri.

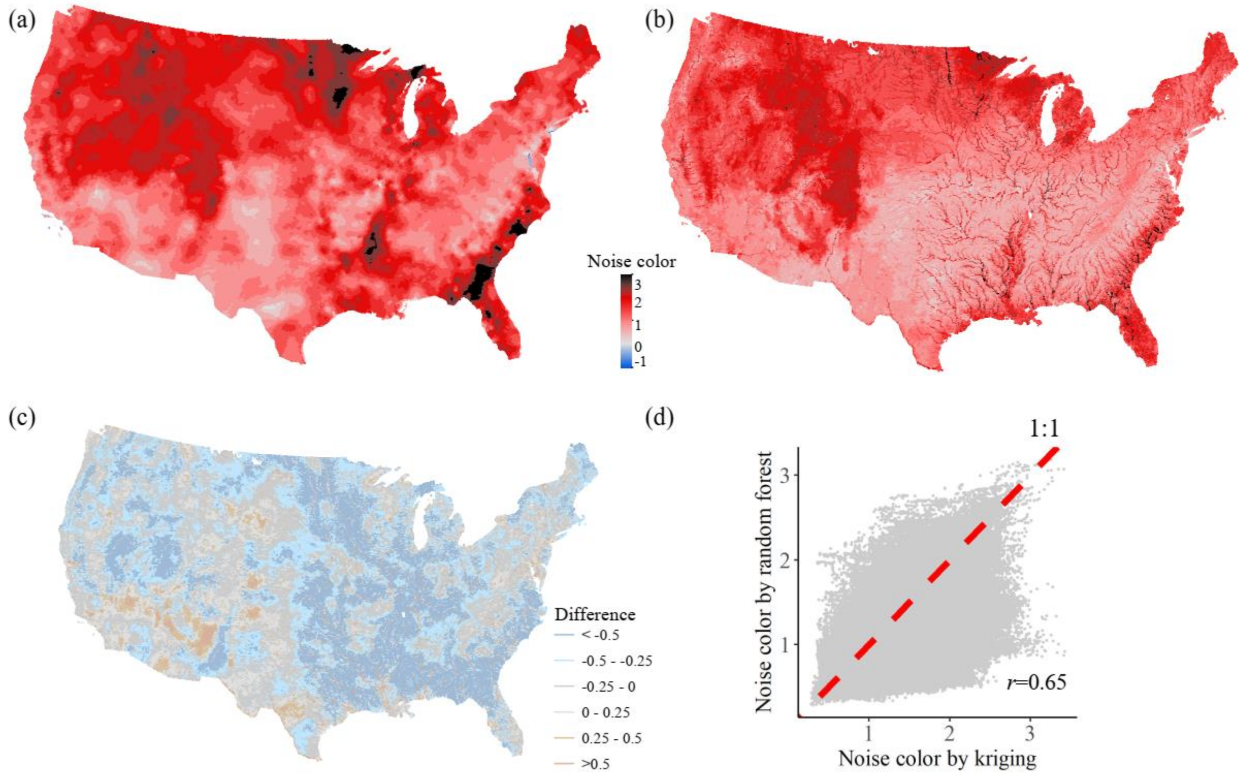

Fig. S12. Comparison of predicted daily noise color across stream segments in the CONUS obtained by (a) spatial interpolation via Empirical Bayesian kriging (EBK), and (b) our Random Forest model (RF). In (c) we map differences in daily noise color between methods, with negative values indicating lower values for RF-predicted than for EBK-interpolated. (d) Comparison between RF and EBK values against a 1:1 red dotted line; each dot is a stream segment (N = 437,766). The contour of the CONUS credits to Copyright:© 2013 National Geographic Society, i-cubed in ArcGIS® software by Esri.

Table S1. Description of the geographic, hydroclimatic, and anthropogenic variables considered in the random forest models, with associated spatial scales and data sources.

Category	Variable Name	Description	Spatial scale	Data source
Geography	Area	Watershed drainage area, sq km	Upstream watershed	1,2,3,4
	Elevation	Elevation, meter	Site	1,2,3,4
Hydro-climate	Stream Order	Stream order (where the main stem is of Order 1)	River reach	1,2,3,4
	Precipitation	Mean annual precipitation, millimeter	Site	3,4,5,6
	Precipitation CV	Coefficient of variation (CV) of mean annual precipitation	Site	3,4,5,6
	Temperature	Mean annual temperature, °C	Site	3,4,5,6
	Temperature CV	CV of mean annual temperature	Site	3,4,5,6
Land use land cover (LULC)	Wetland Cover	Wetland percentage	HUC 10	3,4,6
	Urban Cover	Urbanization percentage	HUC 10	3,4,6
	Forest Cover	Forest percentage	HUC 10	3,4,6
Water management	Water use	Freshwater withdrawal	HUC 10	3,4,6
	DOF	Degree of Fragmentation (details can be found in[1])	River reach	1,3,4
	DOR	Degree of Regulation (details can be found in[1])	River reach	1,3,4

References:

1. Grill, G. *et al.* Mapping the world's free-flowing rivers. *Nature* (2019) doi:10.1038/s41586-019-1111-9.
2. Falcone, J. A. *GAGES II (Geospatial Attributes of Gages for Evaluating Streamflow) summary report*. *Usgs* (2011). https://doi.org/10.3133/70046617
3. Lehner, B. & Grill, G. Global river hydrography and network routing: Baseline data and new approaches to study the world's large river systems. *Hydrol. Process.* (2013) doi:10.1002/hyp.9740.
4. Salas, F. NFIE-Geo Version 2 (20150615). *Hydroshare* (2015).
5. PRISM Climate Group, Oregon State University, https://prism.oregonstate.edu.
6. Falcone, J. A. Changes in anthropogenic influences on streams and rivers in the conterminous U.S. over the last 40 years, derived for 16 data themes. *US Geol. Surv. Data Release* (2018) doi:https://doi.org/10.5066/F7XW4J1J.
