# Supplementary material for: The color of environmental noise in river networks
Source: Nat Commun. 2023 Mar 28;14:1728. doi: 10.1038/s41467-023-37062-2 (PMC10050181; doi:10.1038/s41467-023-37062-2)
Supplement: Supplementary file 2 — Reporting Summary [file 41467_2023_37062_MOESM2_ESM.pdf]

nature portfolio

Corresponding author(s): Tongbi Tu
Last updated by author(s): Feb 21, 2023

Reporting Summary

Nature Portfolio wishes to improve the reproducibility of the work that we publish. This form provides structure for consistency and transparency in reporting. For further information on Nature Portfolio policies, see our Editorial Policies and the Editorial Policy Checklist.

Statistics

For all statistical analyses, confirm that the following items are present in the figure legend, table legend, main text, or Methods section.

n/a	Confirmed	
☐	☒	The exact sample size (n) for each experimental group/condition, given as a discrete number and unit of measurement
☐	☒	A statement on whether measurements were taken from distinct samples or whether the same sample was measured repeatedly
☐	☒	The statistical test(s) used AND whether they are one- or two-sided<br>*Only common tests should be described solely by name; describe more complex techniques in the Methods section.*
☐	☒	A description of all covariates tested
☐	☒	A description of any assumptions or corrections, such as tests of normality and adjustment for multiple comparisons
☐	☒	A full description of the statistical parameters including central tendency (e.g. means) or other basic estimates (e.g. regression coefficient) AND variation (e.g. standard deviation) or associated estimates of uncertainty (e.g. confidence intervals)
☐	☒	For null hypothesis testing, the test statistic (e.g. F, t, r) with confidence intervals, effect sizes, degrees of freedom and P value noted<br>*Give P values as exact values whenever suitable.*
☒	☐	For Bayesian analysis, information on the choice of priors and Markov chain Monte Carlo settings
☒	☐	For hierarchical and complex designs, identification of the appropriate level for tests and full reporting of outcomes
☐	☒	Estimates of effect sizes (e.g. Cohen's d, Pearson's r), indicating how they were calculated

Our web collection on statistics for biologists contains articles on many of the points above.

Software and code

Policy information about availability of computer code

Data collection	The streamflow datasets used in this study are daily streamflow time series from the United States Geological Survey (USGS) flow gages (access at: https://waterdata.usgs.gov/nwis/).
Data analysis	All codes are generated by R (version 4.0.5). The maps are generated by ArcGIS 10.7. The code used in the analysis can be accessed at the DOI: 10.5281/zenodo.7585325.

For manuscripts utilizing custom algorithms or software that are central to the research but not yet described in published literature, software must be made available to editors and reviewers. We strongly encourage code deposition in a community repository (e.g. GitHub). See the Nature Portfolio guidelines for submitting code & software for further information.

Data

Policy information about availability of data

All manuscripts must include a data availability statement. This statement should provide the following information, where applicable:

- Accession codes, unique identifiers, or web links for publicly available datasets
- A description of any restrictions on data availability
- For clinical datasets or third party data, please ensure that the statement adheres to our policy

Data Availability
The datasets generated in this study are available at the DOI:10.6084/m9.figshare.21428061.The streamflow datasets used in this study were retrieved from the

United States Geological Survey (USGS) flow gages (access at: https://waterdata.usgs.gov/nwis/). The precipitation and temperature datasets were retrieved from PRISM Climate Group at https://prism.oregonstate.edu. The water management datasets were retrieved from USGS Data Release at https://doi.org/10.5066/F7XW4J1J. The gage properties were retrieved from USGS at https://doi.org/10.3133/70046617. The datasets of upstream catchment area and stream order were retrieved at https://figshare.com/articles/dataset/Mapping_the_world_s_free-flowing_rivers_data_set_and_technical_documentation/7688801.

Human research participants

Policy information about studies involving human research participants and Sex and Gender in Research.

Reporting on sex and gender	n/a
Population characteristics	n/a
Recruitment	n/a
Ethics oversight	n/a

Note that full information on the approval of the study protocol must also be provided in the manuscript.

Field-specific reporting

Please select the one below that is the best fit for your research. If you are not sure, read the appropriate sections before making your selection.

☐ Life sciences ☐ Behavioural & social sciences ☒ Ecological, evolutionary & environmental sciences

For a reference copy of the document with all sections, see nature.com/documents/nr-reporting-summary-flat.pdf

Ecological, evolutionary & environmental sciences study design

All studies must disclose on these points even when the disclosure is negative.

Study description	In this work, we explore the geography and drivers of flow noise color in river networks. We first examine the spatial patterns of flow noise color at different temporal scales (daily and annual) across streams and rivers in the conterminous United States by leveraging spectral methods on 7,504 gages with long-term high resolution discharge data. We then quantify the associations and relative importance of a suite of natural and anthropogenic variables (geography, hydroclimate, land-use, regulation by dams). Our analysis demonstrates that the characteristics of environmental fluctuations in riverine networks differ from those previously reported from terrestrial and marine ecosystems, and bear the signature of human activities. These results advance current understanding of stochastic variation in the environment—potentially assisting in the identification, management, and conservation of river ecosystems degraded by hydrologic alteration.
Research sample	The streamflow datasets used in this study are daily streamflow time series from the United States Geological Survey (USGS) flow gages (access at: https://waterdata.usgs.gov/nwis/). Gages were selected from all the available gages based on the following criteria: (1) Recording period of at least 15 consecutive years, within 1960 to 2019; (2) missing records being less than 5% of the total length. Any missing data was estimated by linear interpolation. When available, sub-daily flow values were converted to mean daily discharge. Finally, we used daily records from 7,504 gages and annual records from 2,594 gages.<br><br>To investigate the impact of natural and anthropogenic factors, we used the following variables:<br>The precipitation and temperature datasets were retrieved from PRISM Climate Group at https://prism.oregonstate.edu. The water management datasets were retrieved from USGS Data Release at https://doi.org/10.5066/F7XW4J1J. The gage properties were retrieved from USGS at https://doi.org/10.3133/70046617. The datasets of upstream catchment area and stream order were retrieved at https://figshare.com/articles/dataset/Mapping_the_world_s_free-flowing_rivers_data_set_and_technical_documentation/7688801.
Sampling strategy	The sampling frequency is daily (for daily noise color). The frequency of data collection is one of the standard procedures operated by the United States Geological Survey (USGS).
Data collection	The United States Geological Survey (USGS) collected the raw data at streamgages by current meter and Acoustic Doppler Current Profiler when needed.
Timing and spatial scale	At daily and point scale (USGS streamgage locations), across the contiguous United States from 1960 to 2019.
Data exclusions	Following similar methods used in previous studies in handling missing data, gages were selected based on the following criteria: (1) Recording period of at least 15 consecutive years, within 1960 to 2019; (2) missing records being less than 5% of the total length. Any missing data was estimated by linear interpolation. When available, sub-daily flow values were converted to mean daily discharge.
Reproducibility	The results can be reproduced based on the code and data.
Randomization	The randomization was implemented based on a random seed generated by R (version 4.0.5, we provided the random seed in the

codes for reproducibility) and the samples were then randomly split into 80% for training and 20% for test in random forest models. This is a standard procedure in machine learning applications.

Blinding	The study was about streamflow discharge, therefore blinding is irrelevant to our study.

Did the study involve field work? ☐ Yes ☒ No

Reporting for specific materials, systems and methods

We require information from authors about some types of materials, experimental systems and methods used in many studies. Here, indicate whether each material, system or method listed is relevant to your study. If you are not sure if a list item applies to your research, read the appropriate section before selecting a response.

Materials & experimental systems

n/a	Involved in the study
☒	☐ Antibodies
☒	☐ Eukaryotic cell lines
☒	☐ Palaeontology and archaeology
☒	☐ Animals and other organisms
☒	☐ Clinical data
☒	☐ Dual use research of concern

Methods

n/a	Involved in the study
☒	☐ ChIP-seq
☒	☐ Flow cytometry
☒	☐ MRI-based neuroimaging
